# Interplay between Cell-Surface Receptors and Extracellular Matrix in Skin

**DOI:** 10.3390/biom10081170

**Published:** 2020-08-11

**Authors:** Svenja Kleiser, Alexander Nyström

**Affiliations:** 1 Department of Dermatology, Faculty of Medicine and Medical Center, University of Freiburg, Hauptstraße 7, 79104 Freiburg, Germany; 2 Faculty of Biology, University of Freiburg, Schänzlestraße 1, 79104 Freiburg, Germany

**Keywords:** skin, integrin, proteoglycan, syndecan, CD44, TGFβR, EGFR

## Abstract

Skin consists of the epidermis and dermis, which are connected by a specialized basement membrane—the epidermal basement membrane. Both the epidermal basement membrane and the underlying interstitial extracellular matrix (ECM) created by dermal fibroblasts contain distinct network-forming macromolecules. These matrices play various roles in order to maintain skin homeostasis and integrity. Within this complex interplay of cells and matrices, cell surface receptors play essential roles not only for inside-out and outside-in signaling, but also for establishing mechanical and biochemical properties of skin. Already minor modulations of this multifactorial cross-talk can lead to severe and systemic diseases. In this review, major epidermal and dermal cell surface receptors will be addressed with respect to their interactions with matrix components as well as their roles in fibrotic, inflammatory or tumorigenic skin diseases.

## 1. Introduction

Skin consists of two distinct compartments: the superficial epidermis and the dermis below. The epidermis includes the innermost *stratum basale*, *stratum spinosum*, *stratum granulosum* and *stratum corneum* as the outer layer of skin, which is characterized by keratinocytes at progressing differentiation stages, but also involve non-epithelial cells, such as immune cells [1,2]. Additionally, hair follicles and sebaceous and sweat glands are associated appendages of the epidermis [3]. The dermis is subdivided into the upper, papillary dermis and the deep, reticular dermis, which is directly followed by the subcutaneous adipose layer [2]. The dermis also includes blood and lymphatic vessels, nerve endings, hair follicles and sweat glands [1,2]. Even though various immune cells are present in the dermis [1], the major cell type are fibroblasts [4], which produce an interstitial extracellular matrix (ECM) [5]. The ECM orchestrates skin homeostasis, functions as signaling platform and reservoir for soluble factors, and also serves as a structural scaffold that provides both mechanical resilience as well as elasticity [5]. This interstitial ECM consists of collagens, proteoglycans, laminins, fibrillin microfibrils, elastin and matricellular proteins, such as thrombospondins or tenascins [5]. Moreover, a specialized basement membrane—the epidermal basement membrane—exists at the epidermal–dermal junction and builds an anchoring sheet that firmly links the epidermis to the underlying interstitial ECM, while it at the same time functionally divides the epidermis and dermis [6,7,8]. Biochemically, this basement membrane is built in tandem by epidermal keratinocytes and dermal fibroblasts [5].

Both the epidermal basement membrane and the interstitial ECM interact with the cells they tangent or embed in a bidirectional manner in order to create a functioning homeostatic and integer tissue. In this context, a crucial role is assigned to cell surface receptors, since they coordinate cell-signaling events for ECM synthesis, degradation or remodeling. At the same time, they respond to external physical or chemical stimuli and communicate these into cellular responses, such as proliferation, differentiation or migration.

On the basis of these bidirectional cell–matrix interactions in skin, this review will provide a general overview of cell surface receptors in healthy, injured or diseased skin, with a focus on malignancies that involve unremitting inflammation, fibrosis and cancer.

## 2. Integrins

Integrins are the main adhesion proteins that bridge the cellular cytoskeleton with the extracellular matrix (ECM) and thereby serve as bidirectional signal transducers regulating cell proliferation, homeostasis, differentiation, adhesion, migration and apoptosis [9,10,11,12]. The integrin family consists of genetically-distinct alpha and beta subunits that heterodimerize to form functioning transmembrane receptors [10]. In humans, 18 α- and eight β-subunits associate non-covalently into 24 heterodimer pairs [13,14,15]. Each subunit generally comprises a short cytoplasmic region, a single transmembrane segment and a larger ectodomain [14]. After synthesis, both integrin subunits heterodimerize in the endoplasmic reticulum and are subsequently exported to the plasma membrane [16]. Integrins interact with various ECM components, which are recognized either by a specific region on the α-subunit or by motifs on both subunits [17]. Common motifs in the ligands are recognized by groups of integrins such as, e.g., the arginine-glycine-aspartic acid (RGD) in many proteins including thrombospondin-1, tenascin-C, vitronectin, and fibronectin [10,18,19], the leucine-aspartate-valine (LDV) motif in fibronectin [14,20], a triple-helical GFOGER sequence in collagens [14,20,21] and isoleucine-aspartic acid-glycine (IDG) motif of tenascin-C [19]. Importantly, proteolytic cleavage products of ECM components, such as endostatin (from collagen XVIII), endorepellin (from perlecan) or tumstatin (from collagen IV) are ligands and may have similar or different preferences for integrins than the proteins they derive from [20,22,23,24].

At the plasma membrane, integrins undergo conformational changes to transform from an inactive form with low ligand affinity, sometimes over an intermediate form, to a high affinity form to a fully activated ligand-bound integrin [25]. Important for these changes are, in a selection of nine α-subunits, the in-the-ectodomain-inserted von Willebrand factor A domain (I-domain) [10,14,26]. Depending on the ligand bound, the integrin heterodimers regulate different cellular events and literature has presented evidence that at least some integrins select their binding partner in a force- and conformation-dependent manner when they are embedded in a complex ECM that offers various ligands [27,28]. Conversely, the force exerted by the ECM modifies integrin conformation and thus modulates integrin activation, clustering, trafficking and endocytosis as well as various cellular reactions such as proliferation, migration or invasion [17]. Therefore, integrins are widely recognized as mechanoreceptors that translate intra- and extracellular forces into signaling events, which has been reviewed in detail [17,29,30,31,32].

In the classical model of integrin signaling, adaptor and signaling proteins intracellularly cluster around transmembrane integrins to generate focal adhesions, dynamic multi-protein structures that connect the ECM with the actin cytoskeleton [12,33,34]. The focal adhesion proteins including talin, tensin, kindlins and vinculin structurally link the intracellular domain of integrins to the cytoskeletal acto-myosin complexes [35]. Moreover, these proteins are involved in the subsequent recruitment of the focal adhesion kinase (FAK), which is not only a central regulator of focal adhesion (dis-)assembly [36], but also a key player in signaling, since it complexes with Src kinase to be phosphorylated by the latter [37]. This can, in turn, activate various downstream targets and pathways [33,38], for example the PI3K/AKT [34], NFκB [34] or JNK pathway [39] but also cytoskeleton re-organization via Rac1 can be initiated [34].

The main integrins expressed in skin are: α2β1 [40], α3β1 [40] α5β1 [40], α6β4 [40] and αV integrins associating with β3 [41], β5 [40] and β6 subunits [40]. These integrins will be the focus of the review and we will highlight their roles in skin homeostasis as well as selected skin anomalies.

### 2.1. Integrin α6β4

Integrin α6β4 is an integrin rather specialized to epithelial cells. In skin, high abundances of integrin α6β4 subunits are found in basal keratinocytes at their basal plasma membrane adjacent to the basement membrane [40,42,43,44]. The major ligands of integrin α6β4 in skin are the epidermal basement membrane core components laminin-332 and laminin-511 [45].

**Figure 1:**
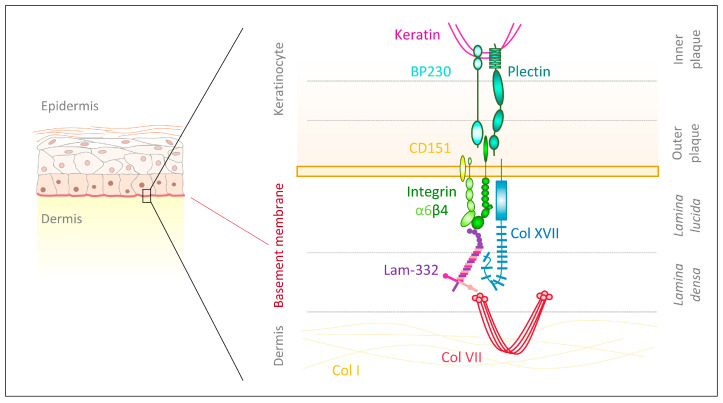
Hemidesmosomal integrin α6β4. Integrin α6β4 is a major component of hemidesmosomes. The cytoplasmic part of integrin β4 is located in the outer plaque close to the plasma membrane and is connected via plectin and bullous pemphigoid antigen 1 isoform e (BPAG1e/PB230) in the inner plaque to the intracellular keratin filaments. Extracellularly, integrin α6β4 binds laminin-332 and CD151 to stabilize hemidesmosomes. Additionally, the transmembrane collagen XVII binds integrin β4, plectin and BPAG1e as well as integrin α6 and laminin-332 in the extracellular space. Laminin-332 further links with collagen VII and together these proteins connect keratinocytes to the underlying basement membrane [51,52].

The integrin β4 differs from other subunits, due to its atypically large cytoplasmic domain containing four type III fibronectin-like repeats [46] and five potential N-glycosylation motifs [47]. The β4 subunit can be phosphorylated through integrin-associated Src family kinase and it subsequently interacts with the adaptor protein Shc and Ras to control ERK and JNK signaling [48]. Additionally, integrin β4 phosphorylation causes activation of MAPK and NF-κB, which foster wound healing and epidermal growth [49,50]. This extended intracellular domain allows for the formation of anchoring complexes—hemidesomsomes—which maintain skin integrity and homeostasis [51]. Type I hemidesmosomes (Figure 1), as they are found in the epidermis, are complexes of integrin α6β4 with intracellular plaques and extracellular anchoring filaments [51]. Within the inner plaque, plectin isoform 1a (P1a) and bullous pemphigoid antigen 1 isoform e (BPAG1e/PB230) link intracellular keratin filaments to the cytoplasmic part of integrin β4, which is located in the outer plaque close to the plasma membrane [51]. Extracellularly, integrin α6 offers binding sites for the tetraspanin CD151 to stabilize hemidesmosomes [51]. Moreover, the transmembrane collagen XVII (BPAG2/BP180) strengthens hemidesmosomes by providing intracellular binding sites for integrin β4, plectin and BPAG1e as well as extracellular docking sites for integrin α6 and laminin-332, though the latter is not sufficient to maintain cellular adhesion [51,52].

Mutations in the *ITGA6* or *ITGB4* gene, encoding for the integrin α6β4 subunits result in the skin blistering disease junctional epidermolysis bullosa (JEB) associated with pyloric atresia (JEB-PA) [53]. Integrin α6β4 deficiency causes aberrant hemidesomsome formation [44,54,55], in the case of *ITGB4* mutations in part through formation of integrin α6β1 [44], which is normally very lowly expressed in basal keratinocytes.

Integrin α6β4 also associates with the receptor tyrosine kinases EGFR family receptors [56,57] through galectin-3-mediated connection of N-glycans [47]. Integrin α6β4-mediated cell adhesion and cell motility are regulated by phosphorylation of the integrin β4 cytoplasmic domain. Serine, tyrosine and threonine phosphorylation of it promotes hemidesmosome disruption [48,57]; conversely, dephosphorylation allows the β4 intracellular domain to associate with the keratin filaments, resulting in hemidesmosome assembly [46,48,58]. It has been reported that laminin-332, which is essential for cell adhesion, inhibits with its short arm of the γ2 chain (γ2sa) EGF-induced phosphorylation of integrin β4 and thereby stabilizes hemidesmosomes [59]. This is regulated by the specific binding of γ2sa to the proteoglycan syndecan-1, which acts as γ2sa receptor on the cell surface and possibly induces signaling cascades that negatively regulate integrin β4 phosphorylation and thus promote stable cell adhesion [59]. On the other hand, the activation of EGFR induces tyrosine phosphorylation of the cytoplasmic integrin β4 subunit through the Src family kinase Fyn [57], further downstream phosphoinositol-3-kinase (PI3K) and ERK are activated to foster cell migration and tumor invasion [57,60,61,62]. In addition, integrin α6β4-mediated PI3K signaling impacts gene expression, for example, the transcription of the integrin α2 subunit or translation of the α3 integrin subunit, which, inter alia, determine migration velocity [63]. Moreover, this signaling controls the translation of genes relevant for (carcinoma) cell survival [64].

Despite being essential for firm, stable cell adhesion evidence supports integrin α6β4 to promote tumor invasion and progression [62,65,66,67,68]. This duality is, in part, enabled through phosphorylation of the integrin β4 subunit’s cytoplasmic domain [69]. Elevated and suprabasal expression of integrin α6β4 is seen in all stages of squamous cell carcinoma (SCC) progression and it has been reported that high suprabasal expression primes SCCs for early relapse [68,70,71,72]. Multiple mechanisms and molecular pathways, including glycan modifications and modulation of the immune microenvironment, underlie integrin α6β4-mediated tumor progression and are extensively reviewed elsewhere [73].

### 2.2. Integrins Containing the β1 Subunit

The integrin subunit β1 pairs with the α1, α2, α3, α4, α5, α6, α7, α8, α9, α10, α11 and αV subunits [10]. The cytoplasmic domain of integrin β1 directly binds various proteins to anchor itself to actin filaments, such as kindlin and talin [74,75,76]. Both proteins keep the integrin in its active form and promote the interaction with the actin cytoskeleton [77,78]. Src family kinases are able to phosphorylate the integrin β1 cytoplasmic tail at two tyrosine residues in the region crucial for talin and kindlin recruitment, which prevents talin and kindlin binding and thereby controls integrin activity [77]. It has been speculated that phosphorylation may modulate integrin signaling such that it initiates transformation and adhesion-independent growth [79,80,81,82].

The major constitutive integrin β1 integrins in skin are in the epidermis α2β1 and α3β1 [40]. After wounding α5β1 and α9β1 can be increased [40]. β1 integrins are also part of specialized niches including integrin α6β1 in hair follicle stem cells [83] and α8β1 in mesenchymal cells in the hair follicle buldge [84].

Integrin α2β1 is in skin found along the lateral and apical surface of basal keratinocytes [85]. It is commonly considered a collagen receptor; however, its ability to bind intact collagen fibrils has been challenged and may rely on fibril-associated proteins [86]. Transmembrane collagen XXIII has been proposed to be an epidermal integrin α2β1 ligand [87]. Many additional proteins bind integrin α2β1 including endorepellin/perlecan [23], which should be considered a major interaction partner at the epidermal basement membrane. Upon collagen interactions, integrin α2β1 lowers cell proliferation but enhances degradation by matrix metalloproteinases (MMP-1, MMP-13) [88,89,90,91,92]. This fosters ECM remodeling and is thought to support the migration of keratinocytes during human wound healing [93]. In contrast, during murine skin wound healing, re-epithelialization, granulation tissue formation and wound contraction by myofibroblasts appear to be independent from integrin α2β1 [94].

Integrin α3β1 localizes at basolateral sites of basal keratinocytes [85], where it is recruited to focal contacts [95] and thereby links keratinocytes to the underlying basement membrane [76]. Integrin α3β1 binds laminin-332 [96] and laminin-511 [97]. Mutations in *ITGA3* encoding the integrin α3 subunit cause congenital nephrotic syndrome, interstitial lung disease, and skin fragility [98], which is classified as a form of junctional epidermolysis bullosa [53]. Both humans and mice with integrin α3β1 deficiency present microblisters at the dermal–epidermal junction with laminin-332 present at both the blister roof and floor [95,98,99].

As integrin α6β4, integrin α3β1 regulates keratinocyte migration. Keratinocytes isolated from integrin α3-deficient mice migrate faster and with increased directional persistence [99], they show elevated stress fiber formation and an accumulation of actin-associated proteins to focal contacts [100]. Additionally, integrin α5β1 and α2β1 activities are enhanced in these cells [100], indicating the β1 subunit to increasingly pair with other α-subunits.

In the epidermis minor integrin, integrin α9β1 promotes re-epithelialization [101].

**Figure 2:**
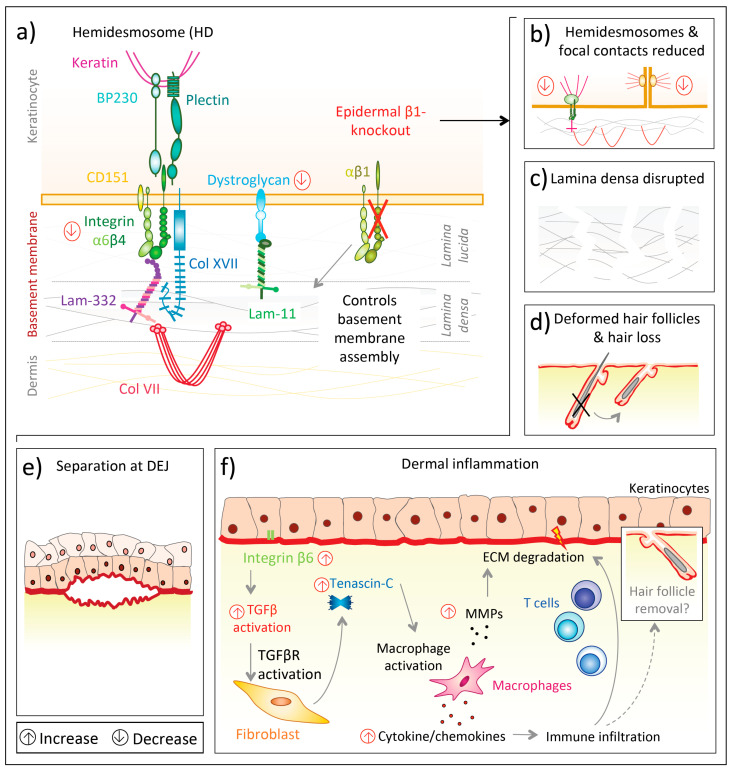
Epidermal integrin β1 deficiency in mice and its consequences. (**a**) Loss of epidermal integrin β1 decreases the laminin receptor dystroglycan and integrin α6β4, which is a major component of hemidesmosomes [102,103]; (**b**) Integrin β1-deficient keratinocytes have fewer hemidesmosomes and focal adhesions [102]. The skin in epidermal integrin β1-deficient in mice presents with diminished lamina densa (**c**) [102], deformed hair follicles resulting in hair loss (**d**) [104] and (**e**) show separation at the dermal–epidermal junction (DEJ) and dermal inflammation (**f**) [105]. A proposed mechanism of the latter is that loss of integrin β1 increases epidermal integrin β6, which is involved in transforming growth factor β (TGFβ) activation. Increased TGFβ signaling upregulates, among others, tenascin-C, fostering dermal cytokine/chemokine production by macrophages, which leads to immune cell (T-cells, mast cells) recruitment. Macrophages also likely cause the increase of matrix metalloproteinases (MMPs) and all together extracellular matrix (ECM) degradation, modified from and inspired by Kurbet et al. [105].

Deficiency of all epidermal β1 integrins is much more severe (Figure 2a) than lack of individual β1 integrins, indicating cooperation and additive effects of them. Keratinocyte-specific integrin β1 deletion in mice under the keratin 5 promoter resulted in severe hair loss as well as mechanically induced skin wounds, though the epidermal barrier function remained stable [102]. In this knockout model, separation at the dermal–epidermal junction was observed, hemidesmosomes were rare (Figure 2b) and the basement membrane was altered, with diminished lamina densa [102] (Figure 2c). Additionally, integrin β1-deficient basal keratinocytes proliferated only weakly and their level of integrin α6β4 was reduced, as was the laminin receptor dystroglycan [102,103] (Figure 2a). Mutant mice displayed thickened epidermis and the authors hypothesized this to be caused by delayed terminal differentiation of suprabasal cells [102]. Moreover, Brakebusch et al. [102] found multiple signs of dermal inflammation. Subsequently, enhanced dermal deposition of collagen I, fibronectin, tenascin-C and perlecan as well as skin stiffening was observed, indicating the presence of dermal fibrogenic processes in mice lacking epidermal integrin β1 [102]. In another mouse model with conditional epidermal integrin β1 deficiency under the keratin 14 promoter [104] newborn mice had a flattened basal epidermal layer and only a thin suprabasal layer before the *stratum corneum*. Additionally, hair follicle development was absent (Figure 2d). Basal keratinocytes from knockout mice proliferated less, though did not prematurely undergo terminal differentiation [104]. Moreover, the basement membrane assembly was compromised, with laminin-332 scattering into the upper dermis and also the abundance of other integrins, such as α6β4, was disturbed or deficient [104]. Accordingly, the back skin of knockout mice was highly fragile and separated at the dermal–epidermal junction upon mechanical challenges [104] (Figure 2e). This separation was possibly a consequence of scarce and morphologically altered hemidesmosomes at the dermal–epidermal junction, as well as a discontinuous lamina densa [104]. Thus, the authors suggested that integrin α6β4, to establish firm hemidesmosomes, requires integrin β1 to control the assembly of an intact basement membrane [104].

The effects on ECM organization and the dermal immune microenvironment upon integrin β1 deficiency have been reinforced by subsequent studies. Kurbet et al. [105] showed that the loss of epidermal integrin β1 disorganizes the basement membrane in early (day E16.5) mouse embryos and progressively causes a sterile inflammation despite an otherwise intact epidermal barrier. (Figure 2f).

β1-containing integrins are in skin not only essential for keratinocytes but also for dermal fibroblasts. Liu et al. [106] found that mice with a fibroblast-specific knockout of integrin β1 had reduced collagen I and αSMA expression and presented a thinned dermis [106,107,108]. This phenotype was in part caused by a reduced Rac1 activation and lowered abundance of reactive oxygen species (ROS) in integrin β1 knockout mice [106]. Moreover, these mice were resistant to bleomycin-evoked dermal fibrosis [108]. The lowered ability of knockout fibroblasts to produce collagen I and αSMA and to differentiate into myofibroblasts also delayed closure of dermal punch wounds and impaired granulation tissue formation and wound contraction in integrin β1-deficient mice [107].

Additionally, dermal fibroblasts from explants of integrin β1 knockout mice showed reduced proliferation as well as migration on collagen I-coated surfaces and also impaired contraction of collagen matrices [107]. Since contractile forces are necessary to activate latent transforming growth factor β (TGFβ), integrin β1-null fibroblasts have a lower ability to activate latent TGFβ [107].

Dermal fibroblasts also express integrin α11β1 as a collagen receptor [109] and upregulate this integrin upon mechanical challenges of the ECM [110]. Integrin α11β1 crucially regulates pro-fibrotic signaling events and also is involved during tissue repair [110]. Accordingly, Schulz et al. [109,111] found that collagen remodeling during skin wound healing is regulated in tandem by integrin α11β1 and non-canonical TGFβ1 signaling. Indeed, wound contraction and granulation tissue formation were diminished in integrin α11β1-deficient mice independent of integrin α2β1. Moreover, these mice presented scar tissue with reduced tensile strength, due to the impaired conversion of dermal fibroblasts into myofibroblasts [109].

In squamous cell carcinomas (SCCs), integrin β1 is required for cell adhesion, spreading and dermal invasion, but, in contrast to normal keratinocytes, not for proliferation [112]. Both of the major epidermal β1 integrins, α2β1 and α3β1, have been investigated in the context of non-melanoma skin cancers. In high-risk cSCCs, arising in the genetic skin blistering disease recessive dystrophic epidermolysis bullosa (RDEB), which is caused by collagen VII deficiency, Martins et al. [113] found that neutralization of integrin α2 with an antibody reduced adhesion of SCC keratinocytes to recombinant human collagen VII, which in turn increased the expression of integrin αVβ6 and TGFβ1 as well as the phosphorylation of Smad2. Thus, they concluded that in cSCC, keratinocyte–collagen VII interaction via integrin α2β1 restrain TGFβ1 signaling [113]. Two-stage chemical carcinogenesis on epidermal integrin α3β1-deficient mice yielded fewer and smaller SCCs compared to wild-type mice [114]. This was explained by enhanced terminal differentiation of α3β1-deficient keratinocytes leading to lower accumulation of mutations in living keratinocytes [114]. Similarly, Meves et al. [79] found the cytoplasmic domain of integrin β1 to endorse skin tumorigenesis independent from its tyrosine phosphorylation status in a Src/FAK-dependent manner that inhibits keratinocyte differentiation. Subsequent to tumor initiation, integrin β1 supports skin tumor invasion and dissemination [115,116,117,118]. Growth factor receptors including EGFR co-operate to facilitate these processes [119,120,121]. Interestingly, the expression levels of EGFR have been shown to depend on matrix attached integrin β1 [122]. EGFR inhibition downregulates integrin β1; vice versa, EGFR activation may stimulate expression of integrin β1 [123]. Combined targeting of EGFR and the integrin β1 subunit has shown promise in preclinical studies to sensitize radioresistant head and neck SCCs to conservative radiotherapy [124].

### 2.3. Integrins Containing the αV Subunit

Integrin αV belongs to the non-I-domain group of α subunits [10] and heterodimerizes with the β1, β3, β5, β6 or β8 subunit [10,12], though it is likely that a hierarchy exists on which β-subunit is preferred for heterodimerization [125].

**Figure 3:**
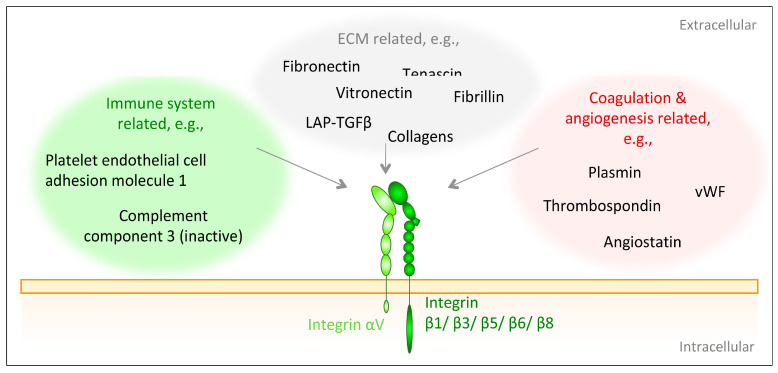
Integrin αV ligands. Integrin αV-containing integrins recognize a multitude of proteins linked to ECM assembly, inflammation or angiogenesis. The selectivity is dependent on the β-subunit [10].

In healthy adult skin, integrin αV is present in the epidermis and dermis, where it reaches its maximal expression levels in the plasma membrane of proliferative basal keratinocytes [33]. It is not restricted to the basal side of keratinocytes but distributed throughout the cell membrane [33]. In the epidermis integrin β5 is the primary heterodimerization partner of integrin αV [126,127]; however, although constitutively present, its abundance is modest [40]. Integrin αVβ6 is found in hair follicle stem cells [40,128] and integrin αVβ8 is expressed in suprabasal epidermal layers in normal skin [40]. Integrin αVβ3 is weakly expressed in healthy skin [41]. Given their low abundance in healthy adult skin, it is reasonable that αV integrins are not required for skin maintenance [33]. After injury, their expression is heavily increased, and they are required for skin regeneration [33]. αV subunit-containing integrins interact with ligands containing an RGD tripeptide motif. Nevertheless, within the integrin αV family, there are differences in the ligand preferences as determined by the heterodimerization partner of integrin αV. Here, integrin αVβ3 displays most promiscuous ligand binding [28] (Figure 3).

To generalize, αV integrins can be viewed as regenerative integrins, and are involved in multiple physiological and pathophysiological regenerative processes. In this context, their ability to activate latent TGFβ1 and 3 is an important trait. This activation occurs through the RGD sequence within the latency associated peptide LAP of TGFβ1 and TGFβ3 [129,130,131]. To be activated by integrin αVβ3, αVβ5, αVβ6 or αVβ8, latent TGFβ has to be also associated with LTBP1 [131,132] or with glycoprotein-A repetitions predominant protein (GARP) [133,134], which is predominantly expressed on T-regulatory cells. LTBP1 localizes and anchors latent TGFβ to the ECM [131,132] while GARP links it to cell surfaces [133]. On the other side, the integrin β-subunits are associated with the cytoskeleton and thereby transmit traction forces from the latter onto the LAP-TGFβ-complex, which ultimately liberates active TGFβ [130,131,132,134]. Additionally, upon binding to latent TGFβ1, integrin αVβ8 is able to simultaneously recruit and bind the membrane type 1 matrix metalloprotease (MT1-MMP), to proteolytically release active TGFβ1 [132,135]. Similarly, integrin αVβ3 is suggested to interact with MMP2 and MMP9 to proteolytically liberate active TGFβ1 [132]. Once TGFβ1 is activated, the β-subunit of integrin αVβ3 complexes with the TGFβRII to control the bioactivity of TGFβ1 as well as to modulate TGFβ1-induced signaling and downstream processes, such as proliferation, ECM deposition or invasion [132,136,137,138].

The integrin-mediated release of active TGFβ is facilitated by increased tissue stiffness [139], which lowers the force needed by the cell to evoke a conformational shift in the ECM-anchored LAP. As TGFβ1 is a pleiotropic fibrotic factor and tissue stiffness is a consequence of fibrosis, a self-perpetuating TGFβ activating loop is created. αVβ3 integrins are the main players of cellular rigidity sensing and cooperate tightly with α5β1 integrins to perceive and react to ECM stiffness [13]. In fibroblasts, integrin α5β1 adheres to fibronectin and creates tension via myosin II activation, while integrin αVβ3 regulates structural adaptations in response to force [13]. In fact, αV integrins cluster at adhesion sites susceptible to high traction forces, but cellular tension due to substrate stiffness is needed to increase the lifetime of fibronectin-αVβ3 integrin complexes. This, in turn, strengthens focal adhesions and induces stress-fiber formation to calibrate cell contractility according to substrate stiffness [13]. Interestingly, data suggest that the mechanical load on the integrin αV-integrin may regulate ligand-binding preferences [28]. Moreover, integrin αVβ3 associates with EGFR, this complex is activated by EGF or fibronectin and subsequently generates paxillin-dependent adhesion and survival signals to prevent anoikis [140].

In terms of its role in cell adhesion, Duperret et al. [33] found integrin αV to associate in large paxillin-containing focal adhesions in fibroblasts, while integrin β1 interacts with smaller focal adhesions. On the other hand, in keratinocytes, integrin β1 is tightly localized with focal adhesions, while integrin αV cannot be detected in a specific subcellular location but spreads throughout the plasma membrane [33]. In this distribution, outside of focal adhesions, integrin αV heterodimerizes with integrins β5 or β6 and together they signal via the focal adhesion kinase (FAK) and the transcription factor c-Myc to control the transition from G1 to S phase in cell cycle, as well as cell proliferation, especially during epidermal tissue generation [33]. Additionally, integrin αV regulates FAK expression, activity and directs it to focal adhesions in keratinocytes [33].

αV integrins are players in wound healing and their roles appear contextual as both overexpression and loss of the same integrin can cause delay of healing [141,142,143]. The various roles of integrin αV during wound healing are reviewed extensively elsewhere [128,144,145]. These integrins are also implicated in the promotion of non-melanoma skin cancer cell migration and invasion. In particular, integrin αVβ6 appears to enhance migration and invasion of SCC cells [146,147], in addition it hampers fibronectin matrix assembly [147] and promotes tumor growth [148].

## 3. Proteoglycans

Proteoglycans are macromolecules containing a core protein with one or more covalently bound glycosaminoglycan (GAG) chains [149]. Either via the protein core or their GAGs, they are able to interact with growth factors and other ECM components to modulate signal transduction, ECM organization and skin architecture [5]. According to their location, they can be classified into intracellular, cell-surface, pericellular and extracellular proteoglycans [150]. In this review, syndecans and CD44 are discussed as cell-surface proteoglycans that function as essential receptors for components of the basement membrane as well as the dermal ECM.

### 3.1. Syndecans

Syndecans are a family of transmembrane proteoglycans and in mammals they comprise four members (syndecan-1, -2, -3, -4) [151] that are present on various epithelial, stromal, endothelial and hematopoietic cells during certain phases of development [152,153,154]. All syndecans consist of an N-terminal extracellular signaling peptide, followed by a transmembrane domain, which also facilitates the dimerization of the protein via a conserved GXXXG motif [155] and thereby assists outside-in-signaling [156,157]. C-terminally, two conserved regions C1 and C2 on the cytoplasmic tail are separated by a variable (v) region that also exerts distinct intracellular roles, such as actin assembly [156,158]. All three intracellular domains carry several serine and tyrosine residues and their phosphorylation status regulates syndecan downstream signaling [159,160,161], which is mediated through binding of intracellular adaptor or scaffold proteins [162,163,164]. For example, the C2 region ends in an EFYA sequence that is able to interacts with PDZ domain proteins [163]. A PDZ domain-containing protein that interacts with syndecans is syntenin-1, which negatively regulates syndecan-4 function [165] and supports syndecan recycling through endosomal compartments [166,167]. The extracellular domain of syndecans harbors several sites for covalent attachment of glycosaminoglycans, which mainly are heparan sulfate (HS) chains. However, syndecan-1 [168,169] and syndecan-4 [170] have been identified as hybrid-type proteoglycans able to carry both HS and chondroitin sulfate (CS) chains [152]. These negatively charged GAGs foster and regulate the binding of cationic extracellular ligands [156,169], among other functions promoting attachment of cells to their surrounding extracellular matrix [171].

**Figure 4:**
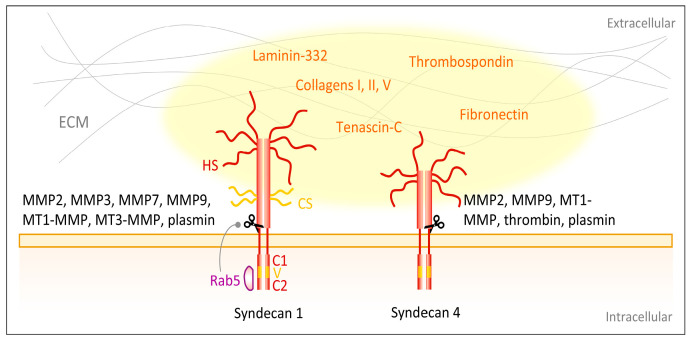
Syndecans interact with ECM components. Major skin syndecans are syndecan-1 and -4. They consist of extracellular signaling peptide containing glycosaminoglycans (GAGs), i.e., heparan sulfate (HS) and chondroitin sulfate (CS) chains, a transmembrane domain and intracellularly two conserved regions C1 and C2 separated by a variable (v) region [155,156,157,158]. Via the GAGs the syndecans interact with ECM ligands, such as laminin-332 [205,206], collagen I, III and V [214]. Additionally, proteins expressed in the transitional ECM of wounds, such as thrombospondin, fibronectin and tenascin-C are ligands [215,216,217,218]. Several proteinases shed the extracellular domain of syndecans, for example matrix metalloproteinases MMP2 [172,173], MMP3 [173], MMP7 [173], MMP-9 [172,173], MT1-MMP [173,174] and MT3-MMP [174] as well as the serine proteinases thrombin and plasmin [173]. The small GTPase Rab5 controls syndecan-1 shedding [176].

Syndecan ectodomains can undergo protease-mediated shedding. Syndecan-1 and syndecan-4 are cleaved by the matrix metalloproteinases MMP-2 [172,173], MMP3 [173], MMP7 [173], MMP-9 [172,173], MT1-MMP [173,174] and MT3-MMP [174], as well as by the serine proteinases thrombin [173] and plasmin [173] (Figure 4). The cleavage sites on the ectodomains of these syndecans are located in close proximity to the plasma membrane [173,175,176,177] and most proteinases recognize and cleave several sites of the core protein [173]. The syndecan protein cores are released together with their GAGs as an entire unit and may be pericellularly retained to compete with plasma membrane-linked syndecans [173]. The shed ectodomains are involved in multiple pathophysiological processes including wound healing [178,179], bacterial and viral pathogenesis [180,181,182] as well as tumor progression [183,184]. Various regulators of syndecan shedding have been identified, such as the HS chains [185]. Reduction of the HS chains increases syndecan-1 shedding [185]. The small GTPase Rab5 has been described to control syndecan-1 shedding, since it specifically binds the cytoplasmic domain of syndecan-1 and dissociation of Rab5 leads to increased shedding [176]. The dissociation can be evoked by binding of the GTPase Rab5 to the cytoplasmic tails of closely interacting integrin β1-subunit-containing integrins [176].

In skin, syndecan-1 and -4 are expressed with high abundances in the epidermis [171,186]. Syndecan-1 is mainly found in the *stratum spinosium* and *granulosum*, and only weakly in basal keratinocytes; it is absent in the *stratum corneum* of intact skin [187,188,189]. On a cellular level, syndecan-1 locates polarized to the basolateral surface of epithelial cells [190,191,192] and is present with high abundance at cell-cell contacts [193]. Human dermal fibroblasts do not constitutively express syndecan-1 [186] and produce only low levels of syndecan-4 [171]. Furthermore, syndecan-2 is not part of healthy adult human skin [194] but is elevated under certain pathological conditions. Its levels are raised in fibrotic dermis, due to the induction of TGFβ and the insulin-like growth factor binding protein-3 (IGFBP-3) [195]. Syndecan-1 is also overexpressed in keloid scars compared to normal or hypertrophic scars [196].

Loss of syndecan-1 expression has also been linked to decreased intercellular adhesion in acantholytic and spongiotic processes, which may foster blister formation in acantholytic or spongiotic dermatosis and also in pemphigus vulgaris or foliaceus [197] and thereby emphasizes an involvement of syndecans in cellular adhesion, skin homeostasis and integrity. In line with this, syndecan-1 and syndecan-4 double knockout mice show increased P-cadherin levels in the epidermal *stratum spinosum* and *stratum basale*, and a disturbed organization of lower epidermal layers, while the suprabasal cells keep their cytoplasmic extensions reaching to the basement membrane [198]. Mechanistically, syndecans interact with transient receptor potential canonical (TRPC) calcium channels and may therefore be involved in regulating actin cytoskeleton, adhesion, junction assembly and cell migration via calcium homeostasis [198,199]. The interaction with TRPCs could also be important in the context of fibrosis. Gopal et al. [198] found syndecan-4 to mediate a myofibroblastic phenotype in primary mouse embryonic fibroblasts via TRPC7. The authors hypothesized that syndecan-4 indirectly interacts with TRPC7, for example via α-actinin, which is known to co-localize with both [198].

Syndecans have been reported to have both pro- and anti-inflammatory effects, most likely depending on the underlying model, the tissue of focus or the stage of inflammation, as well as characteristics of their ectodomains [199]. In the skin disease psoriasiform dermatitis, syndecan-1 has been alluded to an anti-inflammatory function, since it regulates the homeostasis of an interleukin-17-producing subset of γδ-T-cells (Tγδ17) [200].

In wound healing, syndecans may regulate inflammation and cell proliferation. After injury, syndecan-1 becomes highly abundant in keratinocytes at wound margins [187,188,201]. Most likely, TGFβ signaling via protein kinase A (PKA) is responsible for this elevation [202]. A functional role of syndecan-1 in wound healing was established from mouse studies. Syndecan-1-deficient mice showed defective keratinocyte proliferation and differentiation upon wounding [193], as well as decreased keratinocyte migration speed [203]. This may be because syndecan-1 deficiency alters the deposition and assembly as ECM proteins, including laminin-332 and fibrillar collagens, in addition to the cellular interactions with them [203]. It appears as if these events are partially driven by TGFβ1 signaling being constitutively active in syndecan-1-deficient keratinocytes leading to elevated surface abundance of αVβ6, αVβ8 and α6β4 integrins [203]. The altered collagen deposition was speculated to be dependent on the shed ectodomain of syndecan-1 protecting collagen molecules from degradation [203]. Syndecans also support dermal healing, as exemplified by syndecan-4 null mice, presenting with delayed dermal wound healing and diminished angiogenesis [204].

Of specific importance for skin are interactions between syndecan-1 and -4 and laminin-332 [59,205,206]. In a study using normal human keratinocytes, Carulli et al. [205] reported the binding region for both syndecans to lie within the C-terminal globular domains 4 and 5 (LG4-5) domain of the α3 chain of laminin-332; however, the two receptors specifically recognize overlapping but distinct sites and apply discrete binding mechanisms. While binding of syndecan-1 to LG4-5 has been shown to entirely depend on its GAGs [205,206], syndecan-4 also employs its protein core [205]. Upon its secretion and deposition into the basement membrane, laminin-332 rapidly undergoes specific maturation processes, including the cleavage of its LG4-5 domain [207]. The major integrin binding sites are located within the LG1-3 domains but with dependence on the laminin β and γ chain C-termini [208]. Non-processed laminin-332 has been reported to primarily interact with integrin α3β1 and removal of the LG4-5 domain enhances interactions with integrin α6β4 [209]. Syndecan-1 recruitment influences binding and distribution of integrin α3β1, pointing towards an interaction of both receptors [209].

Syndecan-1-mediated cellular adhesion to non-processed laminin-332 retaining LG4-5 has been shown to induce the formation of fascin-containing protrusions via the Rho GTPases Rac1 and cell division control protein Cdc42 [209]. This involves the rapid de-phosphorylation of tyrosine residues in the cytoplasmic regions of syndecan-1 [164] and is proposed to be modulated by the subsequent recruitment and binding of syntenin-1 [164], which in turn has been shown to regulate Cdc42 [210]. Additionally, syndecan-1 or syndecan-4 can complex with the hemidesmosomal integrin α6β4 and ErbB2 or EGFR, respectively, to stimulate migration [211,212,213]. The two syndecans recognize independent sites at the very C-terminus of the β4 integrin and binding critically relies on arginine and glutamic acid, for syndecan-1 and syndecan-4, respectively [212].

Apart from laminin-332, collagen I, III and V [214] and proteins abundant in the transitional ECM of wounded skin such as thrombospondin, fibronectin and tenascin-C [215,216,217] interact with syndecans (Figure 4). Fibronectin binds to the HS GAGs of syndecan-4 with its high affinity heparin-binding domain (HepII) [218] and has been described in several studies to foster cellular adhesion, especially via formation of focal adhesion and actin stress fibers [219,220]. Syndecan-4 is a main receptor for focal adhesion-formation on fibronectin [221,222,223] and has been shown to be recruited into these areas by protein kinase C (PKC) activity [218,223,224]. Additionally, syndecan-4 connects fibronectin with the cytoskeletal component α-actinin [217], providing another link between syndecans and cytoskeletal organization and cellular adhesion. The same binding site on fibronectin that is targeted by syndecan-4, is also recognized by tenascin-C, which therefore interferes with syndecan-4 binding and hampers cellular adhesion [216]. In agreement with this, Midwood et al. [225] showed that syndecan-4-deficient fibroblasts no longer respond to tenascin-C and are therefore able to spread on a fibrin–fibronectin matrix containing tenascin-C, whereas overexpression of syndecan-4 bypasses these inhibitory effects of tenascin-C and normalizes the changes caused by the latter.

Studies indicate syndecan-4 to be involved in mechanotransduction and mechanosignaling [226,227,228]. In fact, mechanical strain increases syndecan-4 expression [229] and during the early stages of mechanical loading, syndean-4 via protein kinase PKCα, activates myosin light chain 2, FAK and ERK [227,228]. In the later stages of mechanical stress, however, these downstream modulators are de-phosphorylated and thus downregulated [227]. Via these cascades, syndecan-4 is indicated to impact actin cytoskeleton assembly, contractility and spreading of epithelial cells [227]. Chronopoulos et al. [226] investigated the role of syndecan-4 as mechanotransducer by locally applying tension force to the receptor itself or its HS chains. This caused EGFR-mediated activation of the phosphoinositide 3-kinase (PI3K) [226]. The latter activated the focal adhesion proteins talin-1 and kindlin-2 and recruited them to focal adhesions, causing the formation of larger and more frequent talin-1 and kindin-2 comprising focal adhesions throughout the cell [226]. Moreover, PI3K activation generated diffusive phosphatidylinositol-3,4,5-triphosphate (PIP3), which interacted with kindlin-2 at focal adhesions and thereby activated integrin β1 [226]. The activated integrin β1-containing integrins, in turn, established new connections to fibronectin and subsequently triggered the activation of the small GTPase RhoA, which finally induced acto-myosin contraction to generate cellular stiffness [226]. Additionally, the application of force onto syndecan-4 strengthened the association of syndecan-4 with α-actinin and F-actin creating a “molecular scaffold” that, through YAP, a well-known mechanosensitive transcription co-activator involved in ECM remodeling [230,231], augmented mechanotransduction [226].

Syndecan-1 in skin seems to protect against cancer initiation and progression. Its loss is associated with transformation of epithelia into anchorage-independent mesenchyme-like cells [232] and also with epithelial malignancies like carcinoma [233]. Accordingly, mice deficient in syndecan-1 present higher conversion of benign papillomas into squamous cell carcinomas than their wild-type peers [189]. Correspondingly, in a study on sporadic and RDEB-associated cutaneous SCCs syndecan-1 was associated with invasion suppression [234]. In this context, MMP-7 expression has been inversely correlated to syndecan-1 abundance, indicating a role of MMP-7 in shedding the protein [234], a function which is beneficial for wound healing [235] but harmful with respect to tumor progression [234]. It should be mentioned that the outcome of syndecan-1 activity on tumor progression could be contextual. Its ectodoamin can regulate activation of integrin αVβ3, which could promote invasion and migration on ECM proteins such as vitronectin [236].

### 3.2. CD44

**Figure 5:**
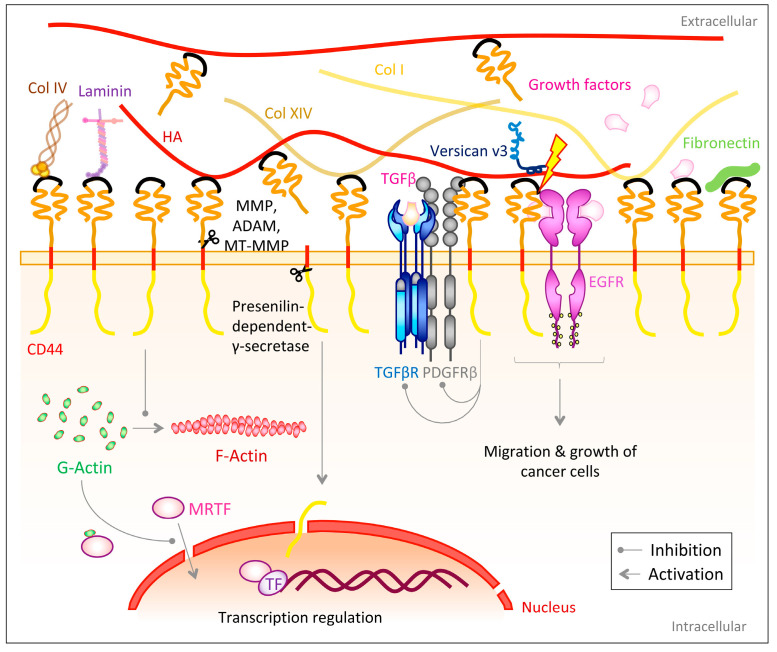
CD44 interacts with various ECM components and cell-surface receptors. CD44 acts as receptor for hyaluronan (HA) [238,239], though can also bind other ECM proteins, such as fibronectin [241], laminin [241], heparin-binding growth factors [245] or collagen I [241], IV [246] and XIV [247]. The extracellular domain of CD44 can be cleaved by MMPs or ADAMs [253,254,255]. They create a soluble form of CD44, which competes with membrane-bound CD44 for HA binding in some cells [256]. The remaining intracellular domain can be released into the cytoplasm by the presenilin-dependent-γ-secretase, where it regulates gene transcription [257,258]. CD44 also prevents the conversion of G- to F-actin. G-actin binds cytoplasmatic myocardin-related transcription factor (MRTF) and hinders it from translocating to the nucleus, where it could co-activate transcription factors (TF) [259]. CD44 interacts with the transforming growth factor β receptor (TGFβR) and the platelet-derived growth factor receptor β (PDGFRβ) and upon HA-activation negatively regulates them [248,249]. CD44 also associates with epidermal growth factor receptor (EGFR) [250,251,252]. Versican possibly competes with CD44 to bind HA and with EGF to bind EGFR and therefore blocks CD44 and EGFR signaling [260].

The transmembrane glycoprotein family CD44 belongs to the group of cell adhesion molecules [237] and is most commonly known as a receptor for hyaluronan (HA) [238,239]. HA is one of the most abundant ECM components in adult human skin and exists both membrane bound as a pericellular coat and freely in the extracellular space [240]. CD44 also serves as binding partner for other ECM components (Figure 5). However, these interactions are only rarely addressed in literature. The ECM proteins fibronectin [241], laminins [241], proteoglycans [242,243,244], heparin-binding growth factors [245] as well as collagen I [241], IV [246] and XIV have been shown to associate with CD44 [247]. Furthermore, CD44 interacts and cross-talks with other cell surface receptors, such as the transforming growth factor β receptor (TGFβR) and the platelet-derived growth factor receptor β (PDGFRβ) by forming a ternary complex, though CD44 is not critical for the interaction of the latter two receptors [248]. Nevertheless, HA-activated CD44 acts as negative regulator on TGFβR and PDGFRβ signaling, probably by recruiting a phosphatase to these growth receptors and/or by destabilizing them [248,249]. Additionally, CD44 has been shown in several studies to interact with the ErbB family of receptor tyrosine kinases and this has been associated with the modulation of tumor cell growth and motility [250,251,252] (Figure 5).

In humans, a single gene on the short arm of chromosome 11 with 19 exons encodes for various isoforms of CD44 [237,239,261]. Of these, the ubiquitously expressed protein, CD44s, represents the smallest isoform and consist of a globularly structured N-terminal extracellular domain with binding sites for its ligands, a transmembrane domain and a C-terminal cytoplasmic domain [237,239,261,262]. However, in the variant isoforms CD44v, the alternative splicing of exons 6–15 creates a variable part, the so-called stem region, which separates the extracellular domain from the transmembrane region [237,239,263]. Furthermore, CD44 can be post-translationally modified by phosphorylation [261] or glycosylation [264] and additionally, its extracellular domain is able to carry heparan sulfate (HS) [245] or chondroitin sulfate (CS) glycosaminoglycan (GAGs) side chains [265], which broadens its repertoire of forms and functions. However, there is limited knowledge about CD44′s specific functions as a proteoglycan [150].

The extracellular domain of CD44 can be proteolytically cleaved by matrix metalloproteinases (MMPs) [266,267], a disintegrin and metalloproteinases (ADAMs) [253] as well as membrane-type-MMPs (MT1-MMP and MT3-MMP) [254,255] (Figure 5), liberating the CD44 extracellular domain as a soluble NH_2_-terminal fragment [257] and thereby allowing the controlled release of cell-surface bound HA [263]. Ectodomain cleavage is regulated by extracellular calcium influx and activation of protein kinase C (PKC) and the small GTPase Rac [253]. On the opposite end after cleavage a membrane-bound COOH-terminal product (CD44EXT), containing the transmembrane and intracellular domain [257] is generated. This remaining domain is subsequently cleaved by the presenilin-dependent-γ-secretase [257,258], which in turn releases the CD44 intracellular domain (CD44ICD) into the cytoplasm. This intramembranous cleavage requires the previous removal of the ectodomain [268]. CD44ICD subsequently translocates to the nucleus to modulate transcriptions dependent on the 12-O-tetradecanoylphorbol 13-acetate (TPA)-responsive element (TRE) [257,268]. Interestingly, one target of this CD44ICD regulated transcription might be the CD44 gene itself, which comprises TRE elements in its promoter region [268].

In skin, the expression patterns of CD44 are independent from gender, age or ethnicity of donors, as well as from the anatomical origin of the sample [269]. However, while dermal fibroblasts mainly express CD44s [269] and only minimal amounts of variable CD44 transcripts [270], 18 distinct and unique transcripts have been identified in epidermal keratinocytes [269]. Moreover, adult keratinocytes display distinct CD44 expression patterns dependent on their differentiation level, with the strongest intensities in the *stratum spinosum* and *stratum basale* [271,272]. An increased expression of CD44 is found in inflamed or neoplastic skin [273,274], both on keratinocytes as well as on infiltrated lymphocytes close to the lesion [273] and is also reported for allergic and irritant contact dermatitis [275].

Although, complete CD44 deficiency in mice does not significantly alter the speed of macroscopic healing of punch biopsy wounds, such mice display alterations of the regeneration of the dermal collagen matrix [238]. During the early phases of wound healing, CD44-deficient mice presented increased inflammatory and reduced fibrogenic responses, such as enhanced leukocyte infiltration but delayed and altered accumulation and spatial distribution of fibroblasts positive for the fibroblast activation protein (FAP) as well as lowered levels of fibrillar collagens [238]. Upon wound closure, however, an accumulation of fibrillar collagens was observed due to a decreased collagen degradation, which promoted severe scarring as well as a lowered tensile strength of the tissue [238].

CD44 knockout mice have been reported to have thinned epidermis with altered differentiation, diminished apical localization of lamellar bodies as well as a delayed recovery of the skin barrier function upon acute perturbation of the *stratum corneum* [276,277], which has been associated with changed expression of tight junction proteins, allocating CD44 a role in tight junction assembly [277]. To specifically address the role of epidermal CD44, Shatirishvili et al. [278] employed a mouse model to, under the control of the keratin 14 promoter, delete CD44 in the epidermis (CD44^Δker^ mice). They observed delayed wound healing, a compromised proliferation and differentiation of keratinocytes as well as a decreased keratinocyte adhesion to and migration on HA coated surfaces [278]. Moreover, atomic force microscopy on skin samples from these CD44^Δker^ mice revealed a reduction in epidermal stiffness, whereas dermal stiffness remained unaffected when compared to wild-type mice [278]. The authors hypothesized that the decrease in epidermal stiffness caused the delayed wound healing properties of these mice and may itself be initiated by a lowered HA production as well as a lack of CD44-dependent HA adhesion [278].

Following the influence of CD44 on dermal healing and ECM deposition, CD44 would also be expected to have effects on fibrosis. Indeed, there is a large volume of studies describing the role of CD44 and its ligand HA in TGFβ-mediated pro-fibrotic signaling; however, findings are contradictory indicating contextuality of CD44 in regulation of fibrogenic processes [259]. While some studies describe CD44 as a supporting and stimulating factor of myofibroblast differentiation and fibrosis [259,279,280], others identify it as an inhibitor of fibrosis and TGFβ signaling [248,259,281,282]. In a murine in vitro model of dermal fibroblasts, Wang et al. [259] identified CD44 as inhibitor of α-SMA gene expression, independent from both the extracellular HA coat as well as HA biosynthesis. Instead, CD44 has been described to, in a yet unknown manner, prevent the conversion of G- to F-actin (actin polymerization) and thereby causing accumulation of G-actin in the cytoplasm. The latter binds cytoplasmatic myocardin-related transcription factor (MRTF) and hinders it from translocating to the nucleus, where it could co-activate the α-SMA transcription factor serum response factor (SRF) (Figure 5) [259].

## 4. Growth Factor Receptors

Growth factor receptors are transmembrane receptors employing protein kinase activity to activate intracellular signaling cascades and thereby modulate, inter alia, cell proliferation, differentiation, metabolism or migration. Prominent players in skin development, homeostasis as well as inflammatory and fibrotic skin malignancies are the transforming growth factor β receptors (TGFβR) and the epidermal growth factor receptor (EGFR). Therefore, and due to their interaction with various ECM components, they are highlighted in this review.

### 4.1. TGFβR

In mammals, there are three transforming growth factor β (TGFβ) isoforms (TGFβ1,-2, -3) [283]; however, the whole family of cytokine genes consists of 33 members [284]. The TGFβ family members signal through receptors (TGFβR) [285], which can be classified into type I (activin receptor-like kinase, ALK) and type II receptors and in humans, seven type I (ALK1–ALK7) and five type II receptors (TGFβRII, ActRII, ActRIIB, AMHRII, BMPRII) exist [285].

Here, with a skin-centric focus, we concentrate on the receptors themselves rather than their ligands. We will refer to them as type I (TGFβRI) or type II receptors (TGFβRII).

The TGFβRs consist N-terminally of small extracellular cysteine-rich domains for ligand binding followed by the transmembrane region and the cytoplasmic kinase domain [286]. TGFβRI additionally holds a regulatory juxtamembrane domain [286]. The receptors assemble into a heterotetrameric complex consisting of two type I and two type II receptors upon TGFβ binding to receptor type II [285,287,288,289,290,291,292]. Subsequently, TGFβRI is recruited and phosphorylated by TGFβRII [285,291,293,294]. This fosters the binding of receptor-regulated Smads (R-Smads) as well as their phosphorylation (i.e., Smad2 and 3 for ALK5 and Smad1, 5 and 8 for ALK1) by TGFβRI [285,295,296,297]. Canonically, ALK5-activated Smad2/3 associate with Smad4 and translocate to the nucleus, where they regulate gene transcription [285,291,293].

Apart from utilizing the canonical Smad signaling pathway, TGFβ is able to regulate signaling and gene transcription via other pathways, such as Ras-ERK-MAPK [293,298,299,300], p38MAPK [301,302], JNK [303,304], PI3K/AKT [305,306], NF-κB [307,308], RhoA [309,310], Rac [311] and Cdc42 [310,311]. Some of these pathways are additionally modulated by betaglycan, which is also referred to as type III TGFβ receptor (TGFβRIII) [312,313]. This transmembrane proteoglycan functions as co-receptor by binding TGFβ and presenting it to TGFβRII, thereby activating downstream signaling cascade [314,315]. Additionally, other transmembrane proteins or ECM components interact and cooperate with TGFβRs to modulate their signaling activities. These include: TGFβRII interaction with the fibronectin receptor, integrin α5β1, at the cell surface, which promotes fibronectin internalization, recycling and incorporation into fibrils in a Smad- and transcription-independent mechanism [316]; TGFβRII clustering with integrin αVβ3 enhancing TGFβ1-induced proliferative effects in the presence of tenascin-C or vitronectin [136]; and direct interaction of integrin α2β1 with TGFβRI and II [317].

In normal human skin, TGFβRI and TGFβRII are expressed in the *strata basale*, *spinosum* and *granulosum* of the epidermis, but can also be found in the dermis, though to a lesser extent [318,319,320]. Additionally, they are highly expressed in sweat and sebaceous glands as well as in hair follicles [320,321,322]. TGFβRII expression is strongly downregulated in aged human dermis [323,324,325].

A broad body of literature on the effects of TGFβ on fibrosis and wound healing exists [326,327,328,329,330,331,332,333,334] and several mouse models [335,336,337,338,339,340] have been developed to study them. However, many of these models present skin anomalies and/or decreased viability [341,342,343], which make them challenging to employ for wound healing studies. Nevertheless, they highlight the role of TGFβ in restricting cell growth and inducing apoptosis in wound healing processes and promoting ECM deposition and remodeling. For example, Liu et al. [335] examined mice with a point mutation in TGFβRI that caused partial receptor activation and found accelerated wound closure and cartilage formation in an ear-punch wound experiment. Mice expressing a dominant-negative TGFβRII mutant exclusively in the basal and suprabasal epidermis displayed thickened, wrinkled skin with a hyperplastic and hyperkeratotic epidermis [336]. Similarly, full-thickness excisional wounds re-epithelialized faster in transgenic mice that express a dominant negative TGFβRII only in keratinocytes [337]. Others created an inducible, fibroblastic TGFβRII knockout model and also found enhanced wound closure, faster re-epithelialization and increased macrophage infiltration [338]. Additionally, collagen deposition and remodeling, wound contraction as well as expression of integrin subunits α1, α2 and β1 were decreased [338]. Similarly, in mice where TGFβRII was deleted postnatally in dermal fibroblasts, excisional wound formation resulted in reduced wound contraction and scarring, while epidermal proliferation was increased [339]. Another fibroblast-selective expression of a kinase-deficient TGFβRII in transgenic mice led to TGFβ hyperactivity with increased fibroblast proliferation, increased ECM production as well as dermal fibrosis [340].

Martins et al. [113] found TGFβRI to be highly expressed in RDEB-associated high-risk SCCs, which occur in a heavily fibrotic microenvironment [344]. Interestingly, this marker was absent or expressed at low levels in non-EB-SCCs [113]. The group also established a stable knockdown of collagen VII in squamous cell carcinoma cells and xenografted 3D cultures onto nude mice. In their system, increased TGFβRI expression in invasive cells within the stroma of xenografts was observed and they suggested a role of TGFβ signaling in RDEB tumorigenesis [113]. Accordingly, Knaup et al. [345] characterized TGFβ1 signaling as architect of RDEB-associated SCC development in a comprehensive gene expression study comparing a non-malignant RDEB keratinocyte cell line to a RDEB SCC keratinocyte cell line.

Cammareri et al. [346] reported mutations in both TGFβRI and II to occur in human sporadic cutaneous SCC samples, while no mutations were detected in distant or perilesional skin. Many of these mutations resulted in a loss of function of canonical Smad signaling, provoking a loss of the TGFβ mediated tumor suppression and the authors grade this as driving event in sporadic cutaneous SCC development [346].

### 4.2. EGFR

The epidermal growth factor (EGF) receptor (EGFR) is a transmembrane protein that belongs to the EGFR family of receptor tyrosine kinases along with that also includes ErbB2, ErbB3, and ErbB4 [347]. In skin, it is expressed both in epidermal keratinocytes [348] as well as dermal fibroblasts [349], though is most highly abundant in proliferative basal epidermal layers [348].

**Figure 6:**
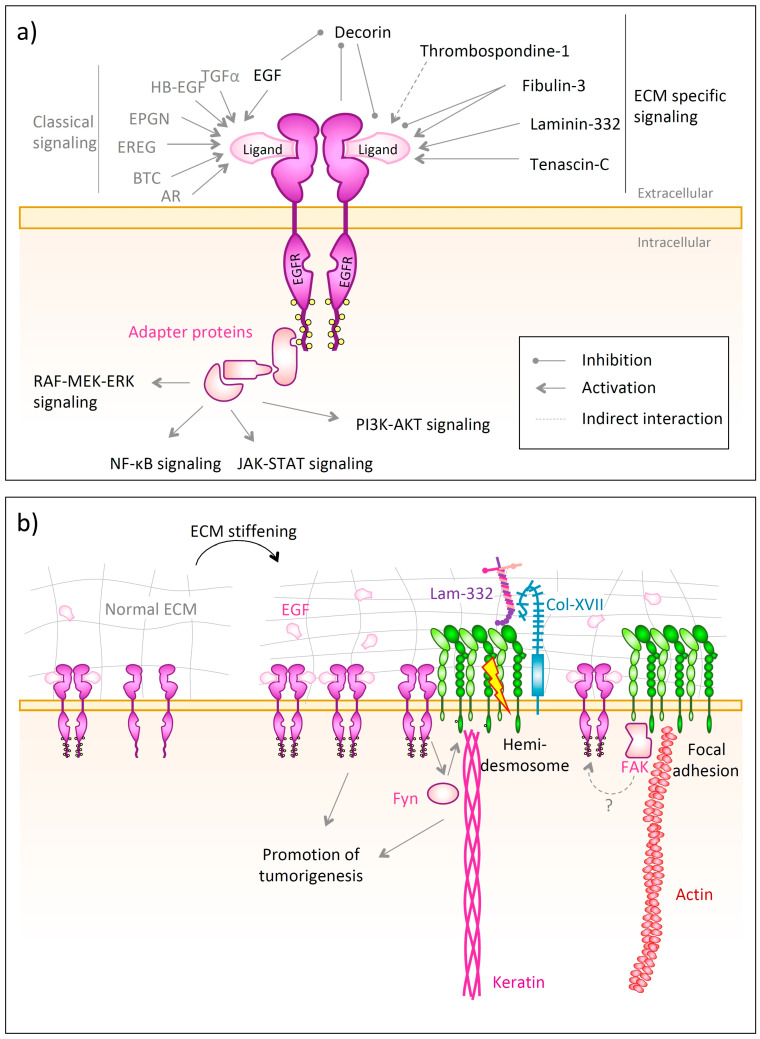
Epidermal growth factor (EGF) receptor (EGFR) interactions with the ECM. (**a**) EGFR recognizes ligands, such as EGF, transforming growth factor (TGF)-α, amphiregulin (AR), betacellulin (BTC), heparin-binding EGF-like growth factor (HB-EGF), epigen (EPGN) or epiregulin (EREG) [352,353]. Upon their binding the receptor dimerizes followed by auto-phosphorylation and induction of diverse intracellular signaling events [351,352,354,355]; (**b**) Stiffening of the ECM, as found in tumors, causes EGFR overexpression and increased EGF bioavailability, which fuels tumorigenesis [359,360,361]. EGFR also associates with integrin α6β4 and via the Fyn kinase phosphorylates β4 integrin if integrin α6β4 is bound to laminin-332 [57,362]. This leads to hemidesmosome disruption and fosters tumor proliferation and invasion [57,362]. Furthermore, a stiff ECM increases EGFR phosphorylation, but this is also dependent on focal adhesions, since EGFR interacts with them and might be regulated by the focal adhesion kinase (FAK) [359].

EGFR consists of an extracellular ligand-binding domain, a transmembrane helix followed intracellularly by a kinase domain and tyrosine-rich C-terminal phosphorylation sites [350,351]. The binding of a ligand, such as EGF, TGF-α, amphiregulin (AR), betacellulin (BTC), heparin-binding EGF-like growth factor (HB-EGF), epigen (EPGN) or epiregulin (EREG) [352,353] to the monomeric extracellular domain causes receptor dimerization and auto-phosphorylation on the intracellular tyrosine residues [351]. This, in turn, allows the docking of adaptor proteins containing Src homology 2 (SH2) or phosphotyrosine binding (PTB) domains [352]. These activate various signaling pathways, such as the Ras-RAF-MEK-ERK pathway [352], the NF-κB cascade [354], the JAK-STAT [355] or the phosphatidylinositol 3-kinase (PI3K)/AKT pathway [352] (Figure 6a). Apart from forming homodimers, EGFR can also heterodimerize with other members of the ErbB family [356]. In order to downregulate EGFR signaling, the receptor undergoes endocytosis and is then sorted to be either recycled to the plasma membrane or to be degraded in the lysosome [357]. One prominent regulator involved in lyososomal degradation of EGFR is the E3 ubiquitin ligase c-Cbl, which binds to active EGFR and then facilitates its poly-ubiquitination and lysosomal degradation [357,358].

Cell–ECM interactions are crucial to maintain cellular metabolic activity by modulating signaling pathways and thereby regulating gene expression or growth factor availability. Detachment of epithelial cells from an ECM causes, inter alia, downregulation of EGFR and integrin β1, both on protein and mRNA level [363]. However, when embedded in an ECM, cells interact with their components utilizing EGFR in a bidirectional manner. For example, ECM composition impacts gene expression in response to EGF stimulation [364], but on the other hand, EGF has been reported to induce the expression of fibronectin in a dose-dependent manner via EGFR signaling [365,366].

Apart from the classical ligands, many ECM proteins influence EGFR signaling and interact with the receptor directly or indirectly. These are decorin, which binds with high affinity to the EGFR [367], causing downregulation of kinase activity and blocking intracellular calcium mobilization and thereby acting as tumor suppressor [367,368,369], tenascin C [370], the γ2 chain of laminin-332 [371], thrombospondin-1 [372] and fibulin-3 [373].

The differentiation and proliferation of epithelial cells is dependent on EGFR signaling. Various studies emphasize the role of EGFR in epidermal development, proliferation and differentiation [374,375,376,377], as well as epithelial motility [378,379,380] and adhesion [381]. This is highlighted by the manifestations of EGFR deficiency in humans. Campbell et al. [382] found an EGFR missense mutation being associated with fragile and highly inflamed skin in a 12 months old infant. While the child was first clinically diagnosed with a subtype of epidermolysis bullosa, it was later specified to carry a mutation located in the extracellular domain of EGFR in a region involved in receptor dimerization. On one hand, this mutation caused unstable EGFR to be rendered at the plasma membrane making it more prone to endocytosis. On the other hand, the mutation also suppressed EGFR phosphorylation and activation of downstream targets, such as ERK or AKT. Clinically, this led to inflamed, frequently infected skin with a reduction in desmosomal proteins as well as alterations in epidermal differentiation. Contrastingly, others have linked EGFR activity with desmosome disassembly and reduced cellular adhesion, whereas the inhibition of EGFR stabilizes desmosomes [383,384,385,386]. In this context, a crucial role is assigned to a disintegrin and metalloproteinase 17 (ADAM17) due to its capability to shed various EGFR ligands [387,388], which fuels EGFR signaling in differentiated keratinocytes. This, in turn, induces protein kinase C and phospholipase C γ1 pathways that terminate in transglutaminase 1 expression [374], which is crucial for crosslinking insoluble proteins of the cornified envelope at the outermost layer of the epidermis [374,389]. Correspondingly, human ADAM17 deficiency due to a loss-of-function mutation within exon 5 of ADAM17 has been associated with epithelial barrier defects resulting in neonatal inflammatory skin and bowel disease [390]. The observed lack of ADAM17 activity possibly prevents various EGFR ligands to be released from the plasma membrane via ectodomain shedding, therefore impeding EGFR activation [391] and corresponding downstream effects.

EGFR relies on the interplay with other receptors for its functions in skin. This is exemplified by interactions with epidermal integrins. It has been shown that a fraction of EGFR directly associates with the hemidesmosomal integrin α6β4 in keratinocytes [57,362] (Figure 6b). Activated EGFR, in turn, activates the Src family kinase Fyn, which phosphorylates the β4 cytoplasmic domain of the integrin. However, this requires the engagement of integrin α6β4 with laminin-332 or its clustering due to specific antibodies [362]. This phosphorylation of the β4 cytoplasmic domain promotes hemidesmosome disruption, which is a requirement for normal keratinocyte migration during wound healing, but also paves the way for squamous carcinoma invasion, proliferation and survival [57,362].

The mechanical properties of tissues are emerging as essential regulators of EGFR activity. Kenny et al. [359] investigated the proliferative response of keratinocytes on tissue stiffness, which was strongly mediated by EGF signaling through EGFR (Figure 6b). In their study, normal human keratinocytes were seeded on model silicone substrates with different elastic moduli coated with either collagen I or fibronectin. The authors revealed the EGFR phosphorylation and activation to be dependent on focal adhesion assembly and cytoskeletal tension. Furthermore, they could correlate EGFR phosphorylation with dermal stiffening in keloid scars, underlining the convolution of biochemistry and biomechanics in scar formation. This is in line with data from Saxena et al. [360], who found EGFR activity to be necessary for rigidity sensing in a model of fibronectin-coated silicone substrates of different stiffness cultured with mouse fibroblasts.

From a pathological perspective, it is known that even moderate stiffening of the ECM sensitizes epithelial cells to EGF, allowing them to proliferate independently of their contact to neighboring cells—a mechanism which is a hallmark of cancer cells [361]. Accordingly, stiffening of the ECM is associated with tumor aggression, metastases and poor clinical outcome in various cancers [392,393]. Likewise, EGFR is frequently overexpressed or strongly activated in several tumor types and fuels tumorigenesis [394,395]. EGFR is also expressed in RDEB-associated SCCs, though levels vary strongly [396,397,398]. Rationally, EGFR inhibition using the monoclonal antibody cetuximab [365] has been tested in single patients with RDEB cSCC and shown some positive but transient effects on lymph node metastases with only mild adverse events [396].

## 5. Outlook

Intensive studies on the receptor-mediated interplay of cells with their surrounding ECM have been carried out during the last few decades, and they have revealed the presence of complex and highly intertwined communication networks that go far beyond simply anchoring cells and matrices together. In fact, these complex systems impact diverse cellular processes, and vice versa, modifications in single cellular receptors may alter the integrity or composition of the entire ECM, promoting systemic diseases. Examples highlighted in this review are mutations of single integrin subunits that result in junctional epidermolysis bullosa, mutations in EGFR that cause skin fragility, or mutations in TGFβRs that impact cutaneous SCCs development. These effects are, in part, also mediated by the tight interplay of cell-surface receptors in skin, such as the direct or indirect interaction of EGFR with syndecans and integrins, which impact each other in a bidirectional manner.

The research work reviewed in this article highlights that malignancies, even though they symptomatically or clinically appear similar, may actually hold distinct underlying mechanisms and should therefore be approached in different ways.

However, an exciting and promising feature of the reviewed diseases is that—despite disease-specific triggers—shared characteristics can be observed not only symptomatically, but also on a biochemical level. These common hallmarks, in turn, may be targeted for therapeutic purposes. A more comprehensive understanding of the bidirectional cell–matrix crosstalks would foster a move from descriptive studies towards the identification and validation of specific therapeutic targets that are common in several conditions. This may pave the way for the development of single therapeutic agents applicable to a broad range of conditions.
